# Molecular docking analysis of PET with MHET

**DOI:** 10.6026/97320630019255

**Published:** 2023-03-31

**Authors:** Omkar D Gowda, Venkatesh CN, Kavitha K, Uday J

**Affiliations:** 1 Shri Vagdevi Educational Trust, 51/3, Behind Vishwa Shanthi Ashrama, Arisinkunte, Nelamangala, Bangalore - 562123; 2 Department of Zoology, Vijayanagar Sri Krishnadevaraya University, Ballari - 583 105, India

**Keywords:** Molecular docking, PET(Polyethylene terephthalate), MHET(monohydroxyethyl terephthalate hydrolase), *Ideonella sakaiensis 201-F6*

## Abstract

An estimated 311 million tons of plastics are produced annually worldwide; 90% of these are derived from petrol. A considerable portion of these plastics is used for packaging (such as drinking bottles), but only ~14% is collected for recycling. Most plastics degrade extremely slowly, thus constituting a major environmental hazard, especially in the oceans, where microplastics are a matter of major concern. One potential solution for this problem is the synthesis of degradable plastics from renewable resources. From the microbial consortium, the researchers isolated a unique bacterium *Ideonella sakaiensis 201-F6* that could almost completely degrade a thin film of PET in a short span of six weeks at 30°C. The objective of the present study is to identify the ligands that may be exploited to improve catalysis and expand substrate specificity and thus significantly advance enzymatic plastic polymer degradation.

## Background:

Plastics are essential materials in our lives because of their advantageous properties such as lightness, durability, low cost, ease of processing into many different forms and non-degradability. However, non-degradability, which was once thought to be a major advantage of using plastics, has been rethought as a major cause of environmental problems, particularly due to the accumulation of waste plastics in landfills and the ocean. Plastics production has steadily increased, with approximately 320 million tonnes of plastics produced globally in 2018 [1]. Because most plastics are difficult to biodegrade and take a long time to degrade, the amount of plastic waste expected to accumulate is expected to reach 33 billion tonnes by 2050 Plastic result, considerable effort has been made to reduce plastic waste. Several chemical degradation methods, such as glycolysis, methanolysis, hydrolysis, aminolysis, and ammonolysis, have been developed to remove plastic waste and recycle plastic-based materials. However, these methods necessitate high temperatures and frequently produce additional environmental pollutants [3]. Biocatalytic degradation, on the other hand, could be used as an environmentally friendly method. Microbes can degrade plastics with ester bonds via enzymatic hydrolysis by colonizing material surfaces. Plastics biodegradability is determined by their chemical and physical properties [4].

The discovery of *Ideonella sakaiensis*could have implications for the degradation of PET plastics. Prior to the discovery of this bacterium, the only known PET degraders were a few bacteria and fungi, such as *Fusarium solani*, and no organisms were definitively known to degrade PET as a primary carbon and energy source. The discovery of *I. sakaiensis* sparked a debate about PET biodegradation as a recycling and bioremediation method [5]. The bacterium was isolated from a symbiotic community of microorganisms in the sediment sample, which also included protozoa and yeast-like cells. It is a gram (-), aerobic, rod-shaped bacteria. It does not produce spores. Cells have a single flagellum and are motile. The bacterium grows best at pH levels ranging from 5.5 to 9.0 (ideally 7 to 7.5) and temperatures ranging from 15 to 42°C (ideally 30-37°C). *I. sakaiensis* colonies are colorless, smooth, and circular. Its width and length range from 0.6-0.8 m and 1.2-1.5 m, respectively. By adhering to the PET and other cells with thin appendages, the bacterium was shown to grow on PET surfaces in a community with other *I. sakaiensis* cells. These appendages may also be responsible for the secretion of PET-degrading enzymes onto the PET surface [5]. The chemical inertness of PET due to the hydrophobicity of the terephthalic acid (TPA) moiety makes it nearly resistant to environmental degradation [6]. After being degraded and absorbed by *I. sakaiensis*, the entire microbial community was shown to mineralize 75% of the degraded PET into carbon dioxide [5].

PET (Polyethylene Terephthalate) is a petroleum-based plastic that is widely used in clothing and plastic bottles. In 2013, approximately 56 million tonnes of PET were produced worldwide, with 15.4 million tonnes for food and liquid containers, 3.2 million tonnes for packaging films, and 38 million tonnes for synthetic fibers [7]. The discovery of *Ideonella sakaiensis*could have implications for the degradation of PET plastics. PETase and MHETase are enzymes that can digest PET plastic polymers found in the bacterium Ideonella sakaiensis. The bacterium's ability to break down polyethylene terephthalate (PET), also known as plastic, using these enzymes has shown great promise in recent years [8]. *Ideonella sakaiensis*cells bind to the PET surface and degrade it into mono(2-hydroxyethyl) terephthalic acid (MHET), a heterodimer of terephthalic acid (TPA) and ethylene glycol, using a secreted PET hydrolase, or PETase. The first PETase was discovered in I. sakaiensis, and it works by hydrolyzing the ester bonds found in PET with high specificity. The resulting MHET is then degraded into its two monomeric constituents by a lipid-anchored MHET hydrolase enzyme, or MHETase [8]. As a result, both of the molecules derived from the PET are used by the cell to produce energy and to build necessary biomolecules. Eventually, the assimilated carbon may be mineralized to carbon dioxide and released into the atmosphere [5]. Because a large portion of manufactured PET is highly crystalline (e.g., plastic bottles), it is believed that any potential applications of the *I. sakaiensis* PETase enzyme in recycling programs will require genetic optimization of the enzyme. In conjunction with the PETase enzyme, the MHETase enzyme could be optimized and used in recycling or bioremediation applications. It converts the MHET generated by PETase into ethylene glycol and terephthalic acid. These two compounds, once formed, can be biodegraded into carbon dioxide by *I. sakaiensis* or other microbes, or purified and used to manufacture new PET in an industrial recycling plant setting [9] PETase, the structure that was solved in 2018, breaks down the plastic into smaller PET building blocks, primarily MHET. MHETase splits this into the two basic precursor building blocks of PET, terephthalic acid and ethylene glycol [10], [11]. MHETase's three-dimensional a rchitecture has some unique characteristics: enzymes like MHETase bind to their target molecule first before undergoing a chemical reaction. An enzyme is required for molecule breakdown. The researchers have already pinpointed where the MHET molecule docks to MHETase and how MHET is then broken down into its two constituents, terephthalic acid and ethylene glycol [12].

## Material and Methods:

## Identification of protein target:

The structural and functional characteristics of proteins such as PETase and MHETase are important to determine the Active Site predictions. Three dimensional structures of these proteins were obtained from RCSB Protein databank such as PET Hydrolase (PDBID: 5XGO withresolution1.58ÅAring;) and MHEThydrolase (PDBID: 6QG9 with resolution of 2.05 Å)

## Protein structure validation and active site prediction:

The validation of 3D modelled structures over stereo chemical quality with residue-by-residue geometry by using Procheck. The residues of loop dictionaries and geometry statistics of non-bonded interactions with different atom types, error functions and plots score were predicted using ERRAT and WHAT_CHECK. Ramachandran plot helps to understand the stereo chemical quality of residue-by-residue geometry, calculated Z-score and volume that predicted the best quality of 3D complex structures. The Ligand binding sites and active sites of 3D modelled protein structures were analyzed by using CastP calculation server [12].

## Ligand preparation:

The binding compounds such as ligand molecules were retrieved from PubChem compound database (http://pubchem.ncbi.nlm.nih.gov/search/search.cgi) 2-Methyl-1,3-pentanediol, 2-Methyl-2,3- pentanediol,2-Methyl-2,4- pentanediol,2-methyl pentane-2,4- pentanediol,4-methoxy 4 methyl -2- pentanol. The ligand optimization was on the basis of phromocore kinetic properties and performed by using Hyperchem Professional 7.0. Identification of related compounds was done by using Lipinski's rule of five. 72 compounds were identified based on similar structure, pharmacore properties and back bone structure 5 compounds were selected. These 5 compounds were optimized by using molecular dynamics and docking was performed.

## Pharmacophore analysis:

The drug-likeness properties along with the pharmacophore and biological activity against different enzymes has been calculated by using Hyperchem 7.5 professional and molinspiration (http://www.molinspiration.com/cgi-/properties), It has used to evaluate drug-likeness and to describe whether a chemical compound has certain pharmacological activity as an orally active drug to human.

## Molecular docking:

Molecular docking studies were carried out using AutoDock 4.2 and AutoDock Tools 1.5.4 from the Scripps Research Institute (http://www.scripps.edu/mb/olson/doc/autodock). The protocol of docking was performed by using methods described earlier by Syed Mohd [14]. The Lamarckian Genetic Algorithm (LGA) was used for ligand conformational searching and the local search algorithm which builds a population of individuals (genes), each being a different random conformation of the docked molecule [15]. The grid was generated around the active site at 80 X 80 X 80 to calculate molecular simulation using AMBER tools, showed auto grid of active site residues around the complex structure. There were 150 populations with mutation rate of 0.02, crossover rate of 0.8 and default grid spacing 0.375 ÅAring; were used as parameter settings for docking. Consequently, these simulations were performed using up to 2.5 million energy evaluations with a maximum of 27,000 generations and each simulation was performed by 10 times, that yielded 10 docked conformations. Finally, the lowest energy conformations were regarded as the binding conformations between ligands and the protein.

## Molecular docking calculations:

Molecular docking of the tetrahedral intermediate from MHETase and PETase structures was carried out by mixed approaches of flexible and covalent docking using AutoDock4.2 [15]. The ligand molecule of PETase was prepared with Ligplot and nonpolar H atoms were merged onto both the ligands and the targets using Auto Dock Tools prior to performing the docking. For the generation of pdbqt files of both rigid and flexible receptor, flexible residues (Tyr87, Trp159, Ser160, Met161, Trp185, Ile208, His237, Ser238, and Asn241) were selected, and the bonds in the side chain of each residue were allowed to rotate. The grid box was centered at x: -3.249, y: 25.239 and z: -29.093 with sizes of 90.7, 74.7, and 122.7Å, respectively. Prior to the covalent docking, non-covalent docking calculation using Auto Dock was performed, and ten output poses were generated with their calculated free energy of binding from its own scoring function. The best docking model with the lowest binding energy was selected, and the conformation of the model was used as an evaluation standard for the following calculation. Furthermore, the induced conformation of the flexible residues in the best model was applied to the receptor for covalent docking. Then, the covalent docking using AutoDock was conducted according to the previous report [16]. A total of 200 docking poses were evaluated based on the proper distances of the oxyanion hole, and the best pose with the binding energy of -10.27 kcal mol-1 (from the semi-empirical free energy force field of AutoDock) was selected by similarity to the non-covalent docking result.

## Results:

## Identification of protein sequence and structure prediction:

The target protein sequences such as PETase are esterase enzyme that catalyzes the hydrolysis of polyethylene terephthalate plastic to monomeric mono-2-hydroxyethyl terephthalate (MHET). The PETase (UID: A0A0K8P6T7) and MHETase (UID: A0A0K8P8E7) sequences were selected from UniProt knowledge database.

## Active Site Prediction BY CASTp Server:

The PETase protein sequence has 290 amino acids with Tyr87, Met161, Trp185 amino acids has substrate binding sites and Ser160, Asp206 and His237 amino acids has active site amino acids have been observed (Table 1). By using CAST P, the main functional Domain region was identified as dienlacteone hydrolase which is in the position between 70 -216. By using CAST P, the main functional Domain region was identified as tannase feuloylestrases which is in the position between 92-600 (Table 2).The MHETase protein also have 600 amino acids of which the active site amino acids were observed in the Ser225, Asp492 and His528 has active site amino acids that binds to acyl-ester intermediate, Asp304, Asp307, Lys309, Asp311, Ile313 amino acids has calcium metal binding region that helps to study the interaction of metal compounds for MHETase inhibition. The protein sequences were used to predict primary structure prediction to understand the physical and chemical parameters of amino acids based on the stability is predicted using Protparam analysis and three-dimensional protein structure is predicted using Swiss model.

The PETase sequence is searched with PSIBLAST Tool using reference protein databank and the results shows 6EQE, 5YFE, 6ILX and 6ILW templates with 100% similar to the target protein structure. MHETase protein sequence also searched with PSIBLAST tool using reference protein databank and the results shows 6QG9, 6QGB and 6QGA templates structures is used to build 3D protein structures. The 3D modeled protein structures is used to predict stereochemical parameters of the residues in the model based on the non-bonded interactions between different atom types and plots using SAVES v5.0 online server.

## Ligand preparation and pharmacological property prediction:

The PTEase and MHETase enzyme hydrolyses active ligand molecules such as bis(2-Hydroxyethyl) Terephthalate is ester of ethylene glycol and terephthalic acid with 2-hydroxyethyl terephthalic acid is an intermediate to produce analogs of target molecule. The ligand structures were used for pharmacophore analysis to predict drug like characters to screen the compounds based on Lipinski Rule of 5 such as hydrogen bond donors <5, hydrogen bond acceptors <10, molecular weight <500kda, topological surface area <140kpa, logP <5 and rotatable bonds <12 bonds are accepted as a best chemical compound. The selected chemical compounds are further used for molecular docking against target proteins using Auto Dock (Table 3).

## Molecular Docking:

The target protein structures such as PETase and MHETase enzymes is docked with bis(2-Hydroxyethyl) Terephthalate derivatives using AutoDock 4.2. The PETase protein is strong docked with 2 compounds having 6 hydrogen bonds having -4.55 kcal/mol of binding energy of which the inhibitory constant of 461.48 µM Kda is binds with active site amino acids. The other compounds such as 1 and 3 also strongly binds with PETase enzyme by forming 3 hydrogen bonds and has binding energy of -5.0 and -3.82 kcal/mol respectively (Table 4).

Another protein MHETase enzyme was also docked with bis (2-Hydroxyethyl) Terephthalate by forming 4 hydrogen bonds having -3.0 kcal/mol of binding energy is strong interacted with active site amino acids. Based on the interaction the 2 compounds showed strong interactions with target molecule and is best used for plastic degradation (Table 5).

## Discussion:

PETase from Idonella sakaiensis was recently found to have significantly higher PET degradation efficiency than the enzymes previously studied. Based on docking calculations, we determined the ligands and reported the structural features conferring high PET-degrading activity on PETase and MHETase. Han et al [17] discovered the crystal structure of PETase and its catalytic mechanism. They used inactive PETase variants instead of wild-type PETase and succeeded in making complex structures of PETase variants (Ser131Ala and Arg103Gly) with two ligands, 1-(2-hydroxyethyl) 4-methyl terephthalate (HEMT) and p-nitrophenol (pNP), respectively, because they failed to obtain complex structures of PETase with various ligands. The substrate-binding mode in complex with HEMT or pNP in the first TPA binding site, which corresponds to the first MHET moiety in this study, is consistent with our findings. We screened 72 compounds for similar structure, pharmacore properties, and backbone structure in this report, and only 5 were chosen. They concentrated on the wobbly tryptophan and serine near the active site due to the complex structure. Because we also observed multi-occupancy of Trp156 (Trp185 in our study), this suggested mechanism indicated by the reduced activity of variant is interesting. On the other hand, we performed docking calculation using a longer substrate which is the major functional domains dienlacteone hydrolase in PETase and tannase feuloylestrases in MHETase. Based on the docking calculation, we thoroughly investigated the substrate binding site by site-directed mutagenesis and concluded that the superior PET-degrading activity of PETase is attributed to the differences in the subsite II and disulfide bond formation. This is an important finding as the structure-based engineering of a residue (Arg280), which is located far away from the catalytic site with a distance of ~ 23 ÅAring; could be selected for enhancing the PETase activity. Based on this study, we propose future studies for PET degradation in the following two directions. First, it will be necessary to characterize PET degradation by other type of enzymes. Second, for actual applications on PET degradation and/or recycling, protein engineering studies toward further enhanced enzyme activity, specificity, and stability are also needed. Also, it is expected that the approaches taken in this study can be extended to studying other enzymes capable of degrading different plastics.

## Conclusion:

The in-silico studies demonstrated that the 2 methyl pentanediol compounds having good binding interaction on PET and MHTE hydrolases. This study reveals the mechanism underlying the catalysis and substrate specificity and thus significantly advances enzymatic plastic polymer degradation. Finally, our results concluded that studying more interactions and validating it experimentally could hopefully provide concepts and solutions for the degradation and recycling of other degradation-resistant plastic materials that are currently used and disposed.

## Figures and Tables

**Table 1:** Showing amino acids positions shown in the table displays decreased enzymatic activity on PET film due to Mutagenesis

**Feature key**	**Description actions**	**Position**	**Length**
Binding site	87	Substrate	1
Active site	160	Charge relay system	1
Binding site	161	Substrate	1
Binding site	185	Substrate	1
Active site	206	Charge relay system	1
Active site	237	Charge relay system	1

**Table 2:** Showing amino acids positions shown in the table displays decreased enzymatic activity on MHET film due to Mutagenesis

**Feature key**	**Description actions**	**Position**	**Length**
Active site	Acyl-ester intermediate	225	1
Metal binding	Calcium	304	1
Metal binding	Calcium	307	1
Metal binding	Calcium	309	1
Metal binding	Calcium	311	1
Metal binding	Calcium;	313	1
Active site	Charge relay system	492	1
Active site	Charge relay system	528	1

**Table 3:** Lignads showing Pharmacokinetic properties, ligands used were 2-Methyl-1,3-pentanediol, 2-Methyl-2,3- pentanediol, 2-Methyl-2,4- pentanediol,2-methyl pentane-2,4- pentanediol,4-methoxy 4 methyl -2- pentanol

**LogP**	**TPSA**	**Natomas**	**MW**	**nON**	**nOHNH**	**Nrotb**	**Vol**
0.49	40.46	8	118.18	2	2	2	128.49
0.66	40.46	8	118.18	2	2	3	129.05
1.11	29.46	9	132.2	2	1	2	146.01
-2.22	43.28	8	117.17	2	1	2	125.74
0.72	40.46	8	118.18	2	2	2	128.49

**Table 4:** Ligands docked with PETase proteins: The protein ligand interaction was calculated based on parameter of H bonds interaction, Binding energy and RMSD Active Sites. The Minimized Free Energy was calculated by from electrostatic and Vander wall interaction between residues of proteins

**Ligand**	**No. H Bonds**	**Bond Energy**	**Inhibitory Constant**	**Amino acids**
1	3	-5	215.48	Asn73, Ala74, Gly75, Lys148
2	6	-4.55	461.48	Pro71, Ala74, Gly75, Gly76, Lys148
3	3	-3.89	1.41mM	Asn275, Ser278, Val281
5	2	-3.45	2.98mM	Cys289, Asn288

**Table 5:** Ligands docked with MHETase proteins: The protein ligand interaction was calculated based on parameter of H bonds interaction, Binding energy and RMSD Active Sites. The Minimized Free Energy was calculated by from electrostatic and Vander wall interaction between residues of proteins

**Ligand**	**No. H Bonds**	**Bond Energy**	**Inhibitory Constant**	**Amino acids**
2	4	-3	6.28mM	Arg101, GLy103,
5	1	-3.64	2.13mM	Gln461, Leu190, Pro186

## References

[1] Joo S (2018). Nature communications..

[2] Rochman CM (2013). Scientific reports..

[3] Gao J (2013). Science signaling,.

[4] Tokiwa Y (2009). International journal of molecular sciences.

[5] Yoshida S (2016). Science.

[6] Palm GJ (2019). Nature communications.

[7] Ji LN (2013). In Applied mechanics and materials.

[8] Palm GJ (2019). Nature communications.

[9] Allam Y (2016). European Spine Journal.

[10] Austin HP (2018). Proceedings of the National Academy of Sciences.

[11] Froimowitz M, Cody V (1993). Journal of medicinal chemistry.

[12] Morris GM, Lim-Wilby M (2008). In Molecular modeling of proteins.

[13] Morris GM (2009). Journal of computational chemistry.

[14] Khan I (2009). Journal of ethnopharmacology.

[15] Morris GM (1998). Molecular docking.

[16] Son HF (2020). Enzyme and Microbial Technology..

[17] Han X (2017). Nature communications..

